# Experimental Study on Flexural Behavior of Simply Supported Beams with All-Light Shale Ceramsite Concrete

**DOI:** 10.3390/ma19163517

**Published:** 2026-08-19

**Authors:** Ran He, Xuyang Zhou, Kun Liu

**Affiliations:** College of Civil Engineering, Hunan City University, Yiyang 413000, China; heran@hncu.edu.cn (R.H.); 2025040027@stu.hncu.edu.cn (K.L.)

**Keywords:** all-light shale ceramsite concrete, simply supported beam, flexural test, material properties, flexural behavior

## Abstract

All-light shale ceramsite concrete (ALSCC) is a lightweight building material suitable for self-weight-sensitive flexural members in prefabricated and long-span structures, but experimental data on its flexural behavior remain limited. This study aims to characterize the physical and mechanical properties of ALSCC and evaluate its flexural behavior under the present test conditions through comparison with C30 normal concrete. Cubic material-property tests and four-point bending tests on simply supported beams were conducted. Material tests showed that the average density of ALSCC was 65.2% of that of normal concrete, while its cube compressive strength and splitting tensile strength were 80.6% and 82.3% of the corresponding values for normal concrete, respectively. Cracks in the ALSCC material specimens tended to propagate through the ceramsite aggregates, indicating a relatively brittle fracture response at the material level. Three ALSCC beams (AL-B1, AL-B2, and AL-B3) and one normal-concrete control beam (NC-B1) were tested to analyze failure modes, load–deflection responses, sectional strain distributions, and reinforcement-strain responses. Test results showed that the ALSCC beams exhibited typical under-reinforced flexural failure, with cracking loads of 5.0–6.5 kN and peak loads of 40.4–42.5 kN, which were comparable to the 41.6 kN peak load of the normal-concrete control beam. The peak loads of the ALSCC beams occurred at midspan deflections of 13.10–14.47 mm. Under continued displacement-controlled loading, maximum recorded midspan deflections of 38.01–40.45 mm were reached at test termination. Within the present test program, the ALSCC beams exhibited lower elastic-stage stiffness than the normal-concrete control beam, while approximately linear sectional strain distributions were observed within the measured load range. The reinforcement-strain responses of the ALSCC beams were also broadly comparable to the response of the control beam. This work provides preliminary material-specific experimental evidence on the flexural behavior of ALSCC simply supported beams under the present test conditions and provides a basis for further validation using larger specimen sets.

## 1. Introduction

Driven by the dual demands of building industrialization, low-carbon development and structural lightweight design, conventional normal-weight concrete has limitations associated with large self-weight and high raw material consumption, which make it difficult to meet the requirements of prefabricated buildings, long-span structures and high-rise frame structures [1,2]. Lightweight-aggregate concrete replaces natural crushed stone with lightweight porous aggregates, which can reduce the structural self-weight by 20–40% while maintaining acceptable mechanical properties. Its thermal-insulation and seismic-performance characteristics have also attracted considerable research attention in civil engineering [3,4]. Existing studies have reported that lightweight-aggregate concrete can reduce the sectional dimensions of structural foundations and members, cut overall project costs, and improve the crack resistance and seismic performance of structures via weight reduction. Its application in green buildings aligns with the national dual-carbon development strategy [5,6].

Shale ceramsite is an artificial lightweight aggregate characterized by moderate porosity, relatively high strength, and favorable interfacial bonding with cement paste. ALSCC is a type of lightweight-aggregate concrete that uses shale ceramsite as the coarse aggregate and shale ceramsite sand as the fine aggregate. Unlike semi-lightweight concrete, in which only the coarse aggregate is replaced by lightweight aggregate, ALSCC uses lightweight aggregates for both the coarse and fine fractions. This composition substantially reduces concrete density and makes ALSCC of interest for self-weight-sensitive structural applications [7,8].

Previous studies have investigated material modification and material-level properties of ceramsite and lightweight concrete, including fiber reinforcement [5,6], nano-silica modification [7], the uniaxial compressive behavior of shale ceramsite concrete [8], mechanical and thermal properties [9], and the effects of lightweight aggregates and steel fibers on concrete performance [10,11]. At the member level, studies have examined composite wall panels containing ceramsite foam concrete [1], the short-term development of cracks in reinforced lightweight-aggregate concrete beams [12], and the flexural behavior of ceramsite concrete beams [13,14,15]. These studies provide an important basis for understanding the material properties and structural responses of lightweight concrete. However, their aggregate systems, mixture proportions, specimen configurations, reinforcement arrangements, and loading conditions differ from those adopted in the present study. In particular, experimental evidence remains relatively limited for beams made with all-light shale ceramsite concrete, in which shale ceramsite and shale ceramsite sand are used as the coarse and fine aggregates, respectively. Therefore, additional material-specific experimental evidence is needed to characterize the flexural response of this concrete system under the test conditions considered in the present study.

Accordingly, the present study provides a material-specific comparative investigation of the flexural behavior of LC30 all-light shale ceramsite concrete beams. Material-property tests and four-point bending tests were conducted on three ALSCC beams and one normal-concrete control beam to examine their failure modes, load–deflection responses, crack development, sectional strain distributions, and reinforcement-strain responses. The contribution of this study lies in providing supplementary experimental evidence for this specific concrete material and specimen configuration, rather than claiming to be the first or a comprehensive investigation of lightweight-concrete flexural members. Considering the limited number of beam specimens, the findings are interpreted as preliminary experimental observations within the scope of the present test program.

## 2. Materials and Methods

### 2.1. Test Materials

**Figure 1:**
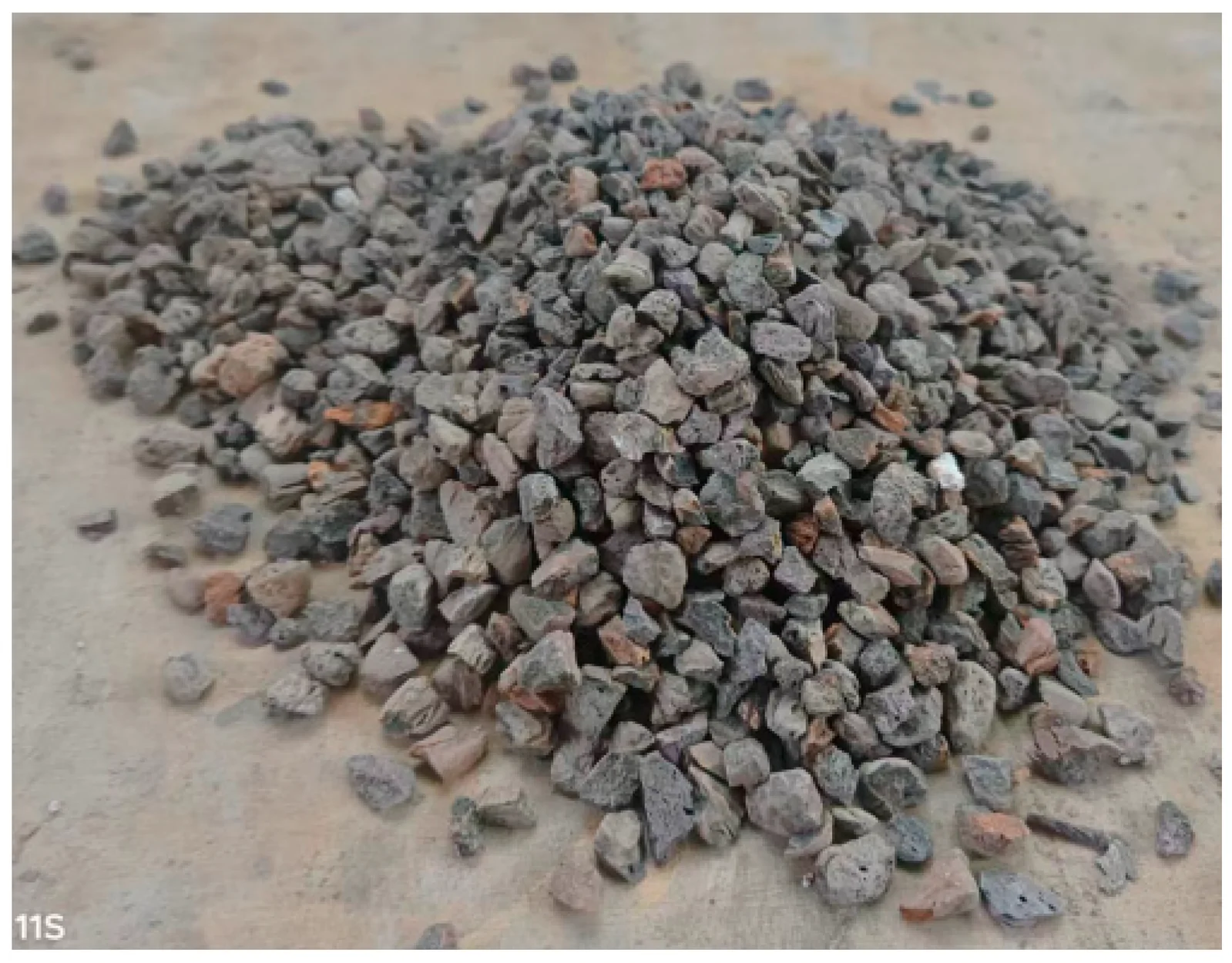
Raw shale ceramsite aggregate.

**Figure 2:**
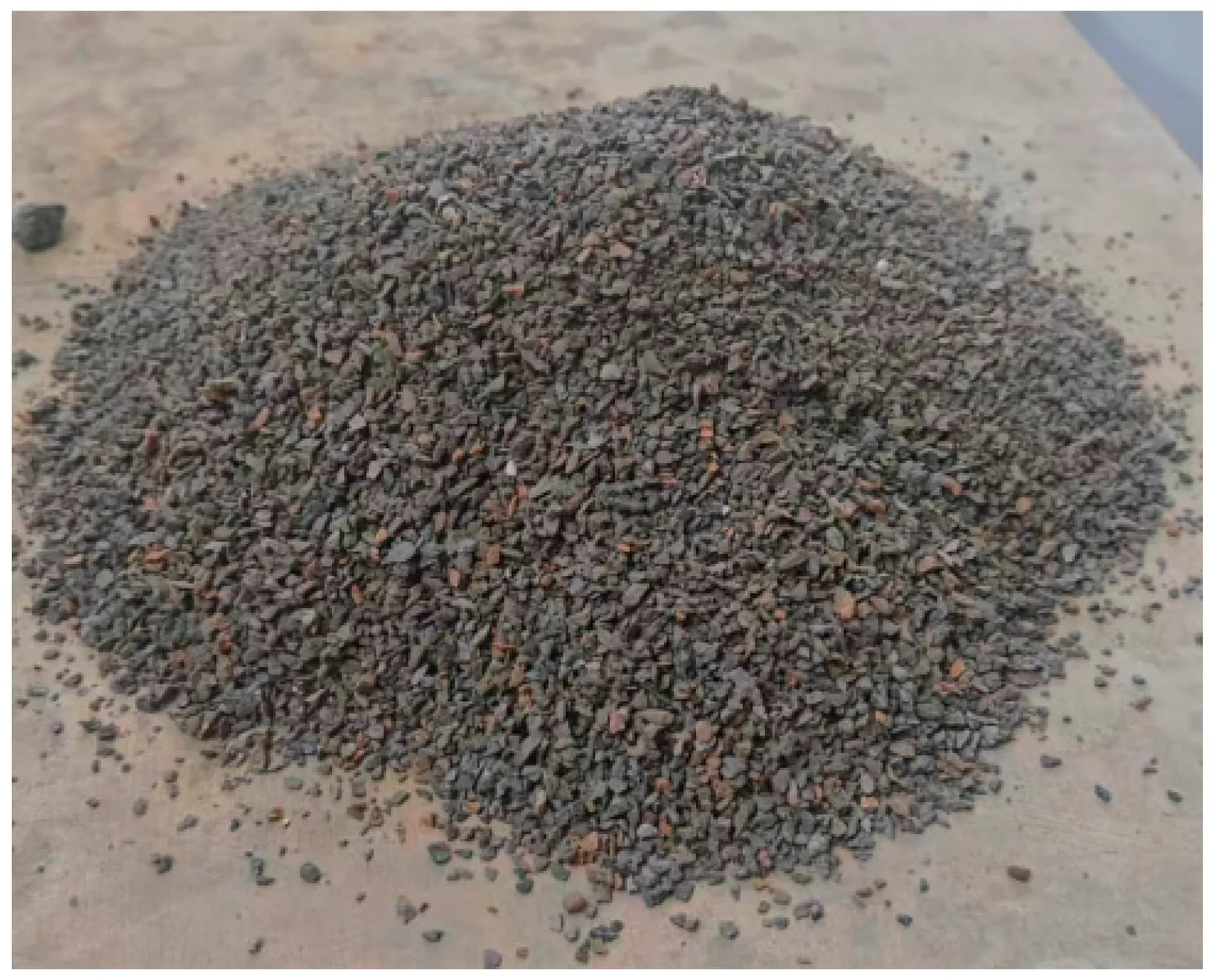
Raw shale ceramsite sand aggregate.

P·O 42.5 ordinary Portland cement was used as the cementitious material in both concrete mixtures. The LC30 ALSCC was prepared using shale ceramsite and shale ceramsite sand as the lightweight coarse and fine aggregates, respectively. Both lightweight aggregates were supplied by Hunan Huaxin Company (Xiangtan, China), and the raw materials are shown in Figure 1 and Figure 2. Cellulose ether and a polycarboxylate-based water-reducing admixture were incorporated into the ALSCC at dosages of 0.3 and 7 kg/m^3^, respectively. The water-to-cement ratio of the ALSCC was 0.54, and its measured slump was 145 mm.

The normal-concrete control specimens were cast using commercially supplied C30 normal concrete, in which 5–20 mm crushed stone and river sand were used as the coarse and fine aggregates, respectively. The C30 concrete contained a polycarboxylate-based water-reducing admixture, had a water-to-cement ratio of 0.50, and exhibited a measured slump of 160 mm. The complete mix proportions and measured slump of both ALSCC and C30 normal concrete are summarized in Table 1.

Two types of steel reinforcement were used in the test, namely HRB400 ribbed steel bars and HPB300 plain round steel bars. HRB400 ribbed bars with a diameter of 12 mm were used as tensile longitudinal reinforcement at the beam bottom. Material tests showed that the measured yield strength and ultimate tensile strength were 430 and 580 MPa, respectively. HPB300 plain round bars were arranged as structural reinforcement at the beam top and stirrups, providing structural confinement and shear resistance for specimens.

The shale ceramsite and shale ceramsite sand were prewetted before mixing.

### 2.2. Material Property Tests

To characterize differences in the physical and mechanical properties of ALSCC and normal concrete, material-property tests were conducted on cube specimens. The cubic specimens and the corresponding test beams were cast at the same time. After demolding, all beam and material-test specimens were cured in the laboratory under natural indoor conditions until 28 days of age. During the curing period, the indoor temperature was approximately 15–20 °C, with a relative humidity of approximately 60–75%. The specimens were sprinkled with water twice daily, generally in the morning and evening, with additional sprinkling when necessary to maintain the concrete surfaces in a moist condition. All test procedures complied with the Standard for Test Methods of Physical and Mechanical Properties of Concrete (GB/T 50081-2019) [16] and the Technical Specification for Lightweight Aggregate Concrete (JGJ 51-2021) [17]. The test data obtained from the cubic specimens provided the fundamental material parameters for the subsequent analysis of the flexural behavior of the beams. The material-property tests in the present experimental program focused on density, cube compressive strength, and splitting tensile strength, while the elastic-stage behavior of the beams was evaluated directly from the measured load–deflection responses.

Separate identification systems were adopted for the material-test specimens and beam specimens to ensure that each specimen had a unique and unambiguous identifier. The normal-concrete cube specimens used for the compressive-strength and splitting-tensile-strength tests were designated NC-C1–NC-C6 and NC-S1–NC-S6, respectively. The corresponding ALSCC specimens were designated AL-C1–AL-C3 and AL-S1–AL-S3, respectively. In these identifiers, “NC” and “AL” denote normal concrete and all-light shale ceramsite concrete, respectively, whereas “C” and “S” denote compressive-strength and splitting-tensile-strength specimens, respectively.

#### 2.2.1. Raw Test Data of Cubic Specimens

The measured density, failure load, and strength of each specimen are summarized in Table 2.

#### 2.2.2. Statistical Analysis of Material Property Test Results

To characterize both the average material properties and the scatter of the experimental data, the mean value, sample standard deviation (SD), coefficient of variation (CV), and 95% confidence interval (CI) were calculated for each material-property group. The number of specimens in each group was also reported, and the statistical results are summarized in Table 3. Combined with the statistical data, the fundamental material characteristics of ALSCC are summarized as follows:

(1) Lower density: The average density of ALSCC was 65.2% of that of normal concrete, demonstrating a substantial reduction in material density.
(2) Strength relative to normal concrete: The cube compressive strength and splitting tensile strength of ALSCC were 80.6% and 82.3% of those of normal concrete, respectively. Thus, the relative reductions in strength were smaller than the reduction in density. In addition, the splitting-tensile-strength-to-compressive-strength ratios were approximately 8.5% for ALSCC and 8.3% for normal concrete, respectively. Given the marginal difference of approximately 0.2 percentage points, the two ratios were considered broadly comparable.
(3) Brittle fracture tendency at the material level: Cracks in the ALSCC material specimens tended to initiate and propagate through the ceramsite aggregates rather than along the aggregate–mortar interfacial transition zone. This crack path indicates a relatively brittle fracture response of ALSCC at the material level.

**Table 3:** Statistical results of the physical and mechanical properties of concrete.

Concrete Type	Property	*n*	Mean	SD	CV (%)	95% CI
Normal concrete	Density (kg/m^3^)	12	2305.34	35.02	1.52	2283.09–2327.58
Normal concrete	Cube compressive strength (MPa)	6	38.08	2.94	7.73	34.99–41.17
Normal concrete	Splitting tensile strength (MPa)	6	3.16	0.34	10.60	2.81–3.52
ALSCC	Density (kg/m^3^)	6	1503.66	20.01	1.33	1482.65–1524.66
ALSCC	Cube compressive strength (MPa)	3	30.68	2.13	6.93	25.40–35.96
ALSCC	Splitting tensile strength (MPa)	3	2.60	0.21	7.95	2.09–3.11

The coefficients of variation in cube compressive strength were 7.73% and 6.93% for normal concrete and ALSCC, respectively, while those of splitting tensile strength were 10.60% and 7.95%. The relatively wide confidence intervals of the ALSCC strength groups should be interpreted with caution because only three specimens were tested in each group.

### 2.3. Specimen Design

A total of four simply supported beams were designed in this test. The three ALSCC beams were designated AL-B1, AL-B2, and AL-B3, whereas the normal-concrete control beam was designated NC-B1. In the specimen identifiers, “B” denotes a beam specimen. The beam geometry and reinforcement arrangement were kept identical, while the concrete type was varied. It should be noted that the two concretes differed in their measured density, cube compressive strength, and splitting tensile strength. Therefore, the observed differences in beam response should be interpreted in conjunction with the measured material properties rather than being attributed solely to aggregate type. All specimens adopted identical cross-sectional dimensions and reinforcement schemes: the cross-section was 100 mm × 180 mm, the span between the supports was 2.0 m, and the total beam length was 2.2 m. A 100 mm end overhang was provided beyond each support to accommodate reinforcement anchorage.

**Figure 3:**
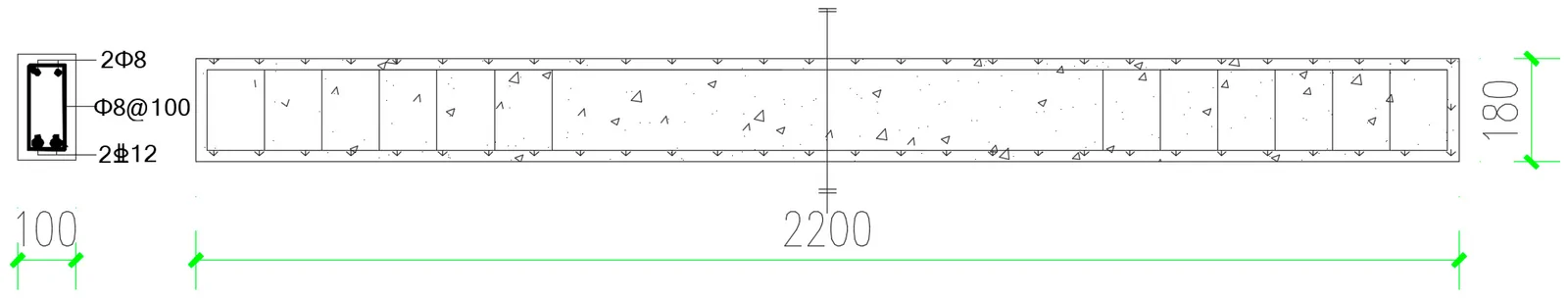
Dimension and Reinforcement Drawing of Test Beams.

In terms of reinforcement arrangement, two 12 mm diameter HRB400 ribbed bars were used as the bottom longitudinal tensile reinforcement. HPB300 plain round steel bars were adopted for both top structural reinforcement and stirrups. The top structural reinforcement was 2Φ8, which functioned to fix stirrups and resist shrinkage stress of the beam. The stirrups had a diameter of 8 mm with a spacing of 100 mm, arranged as two-legged stirrups to restrain the longitudinal bars and provide shear resistance. The concrete cover thickness for all steel reinforcements was controlled at 20 mm. The overall dimensions and reinforcement details of the specimens are illustrated in Figure 3.

### 2.4. Test Loading Scheme

**Figure 4:**
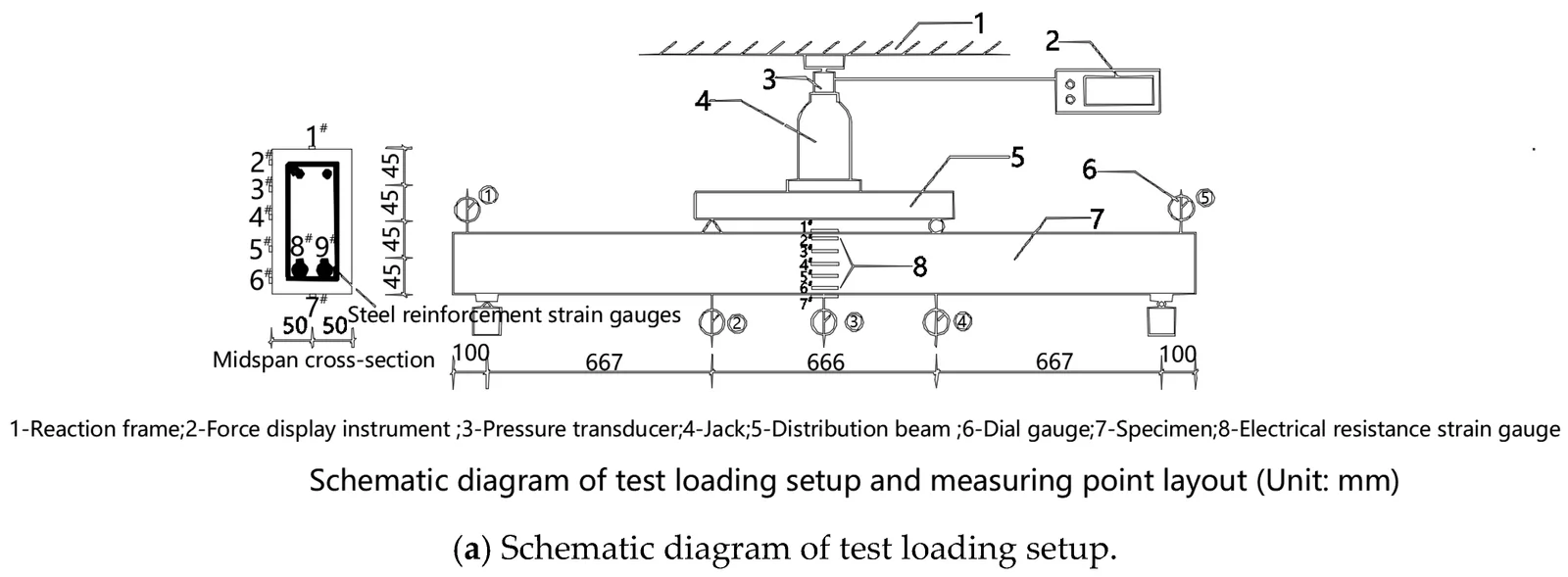

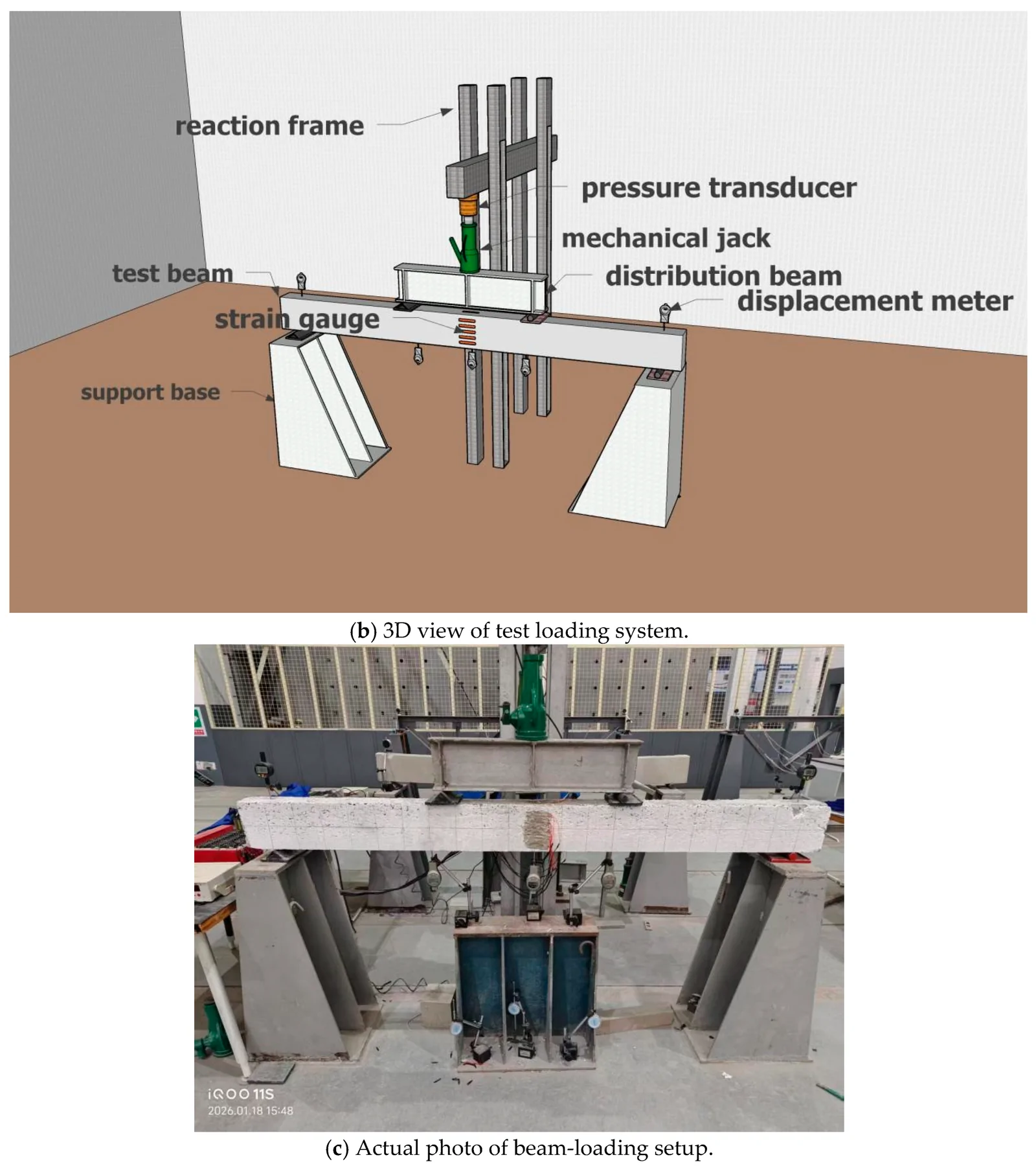
Four-point bending test setup and instrumentation arrangement.

Four-point bending was adopted for all beam tests. The applied load was transferred through a distribution beam to two symmetrically positioned loading points on the specimen. Each loading point was located approximately 667 mm from the adjacent support, and the spacing between the two loading points was approximately 666 mm, thereby forming a constant-moment region between them. The loading setup consisted of a reaction frame, hydraulic jack, pressure transducer, and distribution beam, as illustrated in Figure 4. Figure 4a shows the loading and measurement layout, Figure 4b presents the 3D view of the loading system, and Figure 4c shows the actual beam-loading setup. The measurement contents and loading procedures are described as follows:

#### 2.4.1. Measurement Point Layout

Displacement meters were arranged at the two loading points, the supports, and midspan to measure beam deflection. Five concrete strain gauges were bonded along the depth of the midspan section at 30 mm intervals. In addition, one strain gauge was attached to each of the top and bottom surfaces to record the surface strains. Two steel strain gauges were bonded to the bottom tensile longitudinal reinforcement within the constant-moment region to record the reinforcement-strain response.

#### 2.4.2. Preloading

A preload of 1 kN was applied and maintained for 5 min before formal loading. This step was used to verify the operation of the strain-acquisition system and displacement gauges and to ensure proper contact between the beam and the supports. The beam was then unloaded to zero.

#### 2.4.3. Formal Loading

A staged loading protocol was implemented. Before concrete cracking, each load increment was 0.5 kN with a holding time of 3 min to observe crack initiation. The load corresponding to the first visible crack was defined as the cracking load. Afterwards, the load increment was adjusted to 4 kN with a holding time of 5 min, during which the development and distribution of visible cracks were observed and documented. When the steel strain reached the yield strain and the load growth slowed down, steel yielding was identified, and the loading mode was switched to displacement control with a displacement increment of 3 mm per stage. The test was terminated when severe crushing and spalling occurred in the concrete compression zone at the beam top.

### 2.5. Test Phenomena

**Figure 5:**
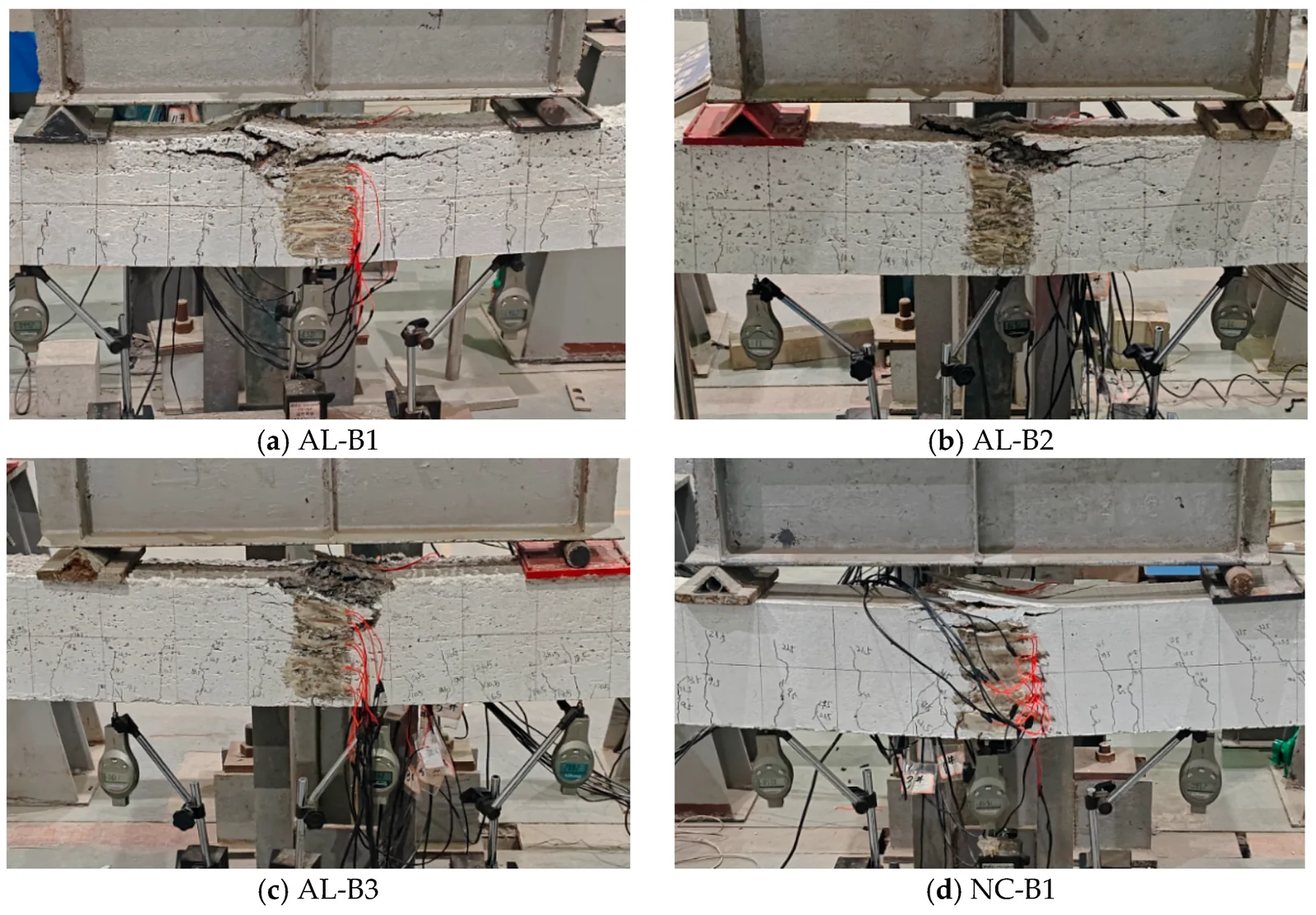
Failure Patterns of Test Beams.

All four test beams exhibited typical under-reinforced flexural failure, characterized by yielding of the tensile reinforcement followed by crushing of the concrete in the compression zone. The overall failure process comprised four stages: elastic, cracking, post-cracking, and failure. Although the overall failure mode was similar among the tested beams, differences were observed in crack propagation and local failure morphology. The main test observations are described below. The failure patterns of all test beams are shown in Figure 5.

In the elastic stage, the beams deformed without visible cracking, and the measured reinforcement and concrete strains increased approximately linearly with load. Both beam types exhibited stable elastic responses before cracking; the quantitative comparison of elastic-stage stiffness is presented in Section 3.1.

As the load increased, the test beams entered the cracking stage, and the first vertical crack appeared within the constant-moment region. The cracking load of ALSCC beams ranged from 5 kN to 6.5 kN, while that of NC-B1 was 5.5 kN. Cracks in ALSCC beams mostly originated inside ceramsite aggregates, whereas cracks in NC-B1 initiated near the aggregate–mortar interface. The deflection increased more rapidly after cracking, and an inflection point appeared in the load–midspan deflection curve, marking the transition from the elastic stage to the post-cracking stage.

**Figure 6:**
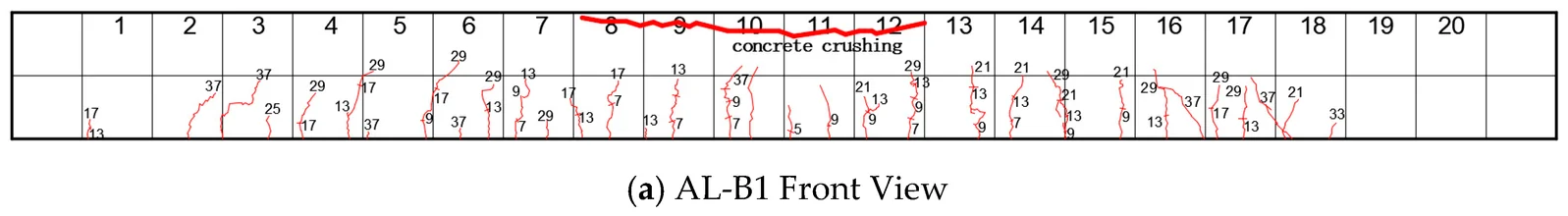

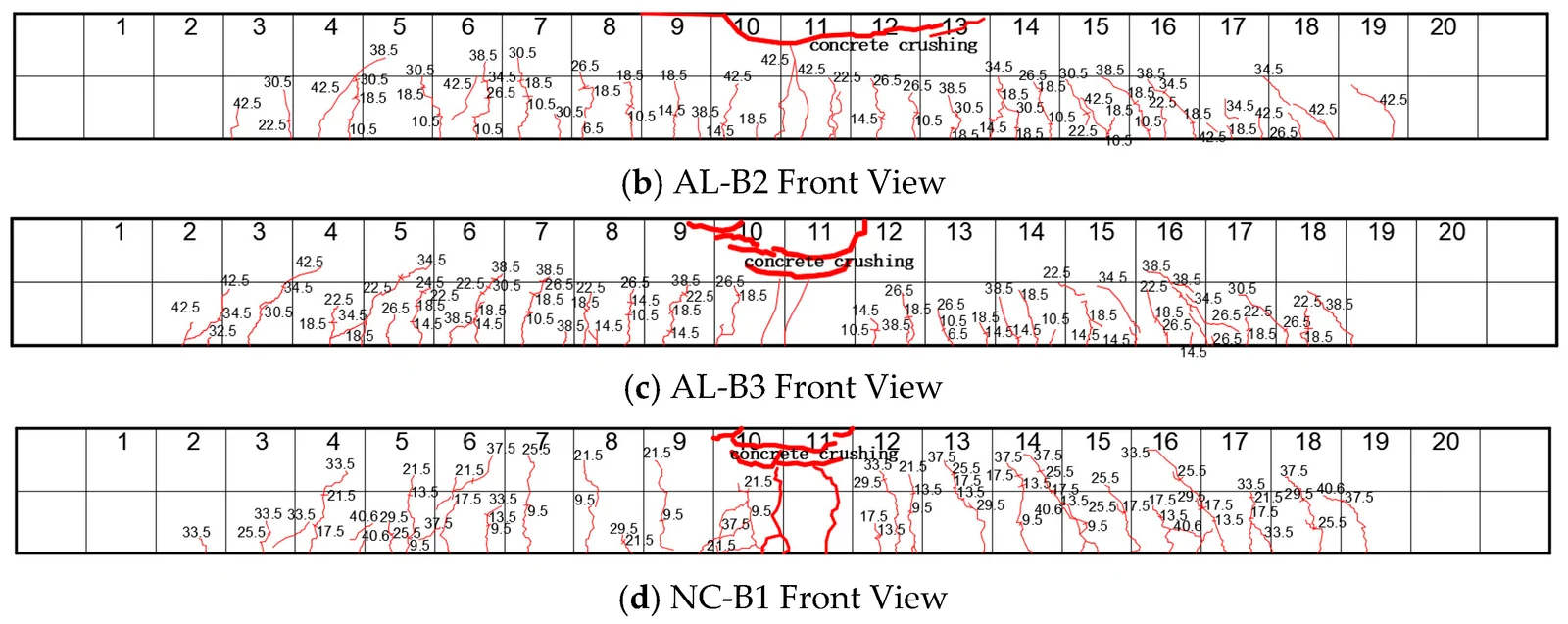
Crack Distribution Diagrams of All Test Beams. Note: The values marked beside cracks represent the concentrated load (unit: kN) recorded when corresponding cracks formed during the test.

During the post-cracking stage, the reinforcement strain continued to increase and gradually approached yielding, while the deflection growth rate increased. Visible cracks continued to develop in the constant-moment and flexure-shear regions. The ALSCC beams generally exhibited a relatively distributed crack pattern, with vertical flexural cracks developing mainly in the constant-moment region and short diagonal cracks also observed in the flexure-shear regions. In comparison, NC-B1 showed a comparatively more localized crack-development pattern. Slight cracking sounds of broken ceramsite particles were captured during loading on ALSCC beams, while crisp cracking noises from concrete interfacial debonding were heard for normal concrete beams. Such acoustic differences reflected the distinct internal force transfer mechanisms of the two materials. The corresponding crack distributions of all test beams are shown in Figure 6.

As the load approached the peak load, the beams entered the failure stage. The rate of load increase decreased, while the midspan deflection increased rapidly and cracks propagated toward the compression zone at the beam top. The peak loads of the ALSCC beams ranged from 40.4 to 42.5 kN, which were close to the 41.6 kN peak load of the normal-concrete control beam. AL-B2 and AL-B3 exhibited slightly higher peak loads in the present tests. Finally, the concrete in the compression zone was crushed and spalled. Local block spalling occurred in the compression zone of ALSCC beams, whereas NC-B1 exhibited a wider crushing region.

Overall, the ALSCC beams and NC-B1 exhibited the same under-reinforced flexural failure sequence, although differences were observed in their crack patterns and local failure morphology. Such differences essentially stem from the distinct internal crack initiation mechanisms and propagation laws of the two concrete materials.

The interfacial bonding between mortar and ceramsite aggregates in ALSCC is compact, and the mortar strength exceeds that of ceramsite aggregates. Therefore, cracks preferentially initiate inside ceramsite aggregates and extend into the mortar matrix. After cracking, numerous fine, uniformly distributed cracks with small load increments form in the pure bending and flexure-shear segments, without obvious dominant main cracks. In contrast, natural aggregates possess higher strength than mortar in normal concrete, so cracks germinate at weak aggregate–mortar interfacial zones and rapidly propagate along such weak planes after cracking. This generates fewer, longer cracks with larger load increments, which are concentrated within the constant-moment region.

The divergent crack development characteristics directly lead to distinct failure details. Only local block spalling occurs in the compression zone of ALSCC beams with a small crushing area, whereas normal concrete beams exhibit a wider crushing range and more prominent local collapse features in the compression zone. Although cracking through ceramsite aggregates indicates a relatively brittle response at the material level, this did not result in premature brittle flexural failure of the reinforced beams in the present tests. All tested beams exhibited an under-reinforced flexural failure sequence, with yielding of the tensile reinforcement preceding crushing of the compression-zone concrete. The relatively distributed crack pattern observed in the ALSCC beams is therefore considered a crack-morphology characteristic of the tested specimens rather than direct evidence of improved member ductility.

## 3. Results

### 3.1. Analysis of Load–Deflection Curves

**Figure 7:**
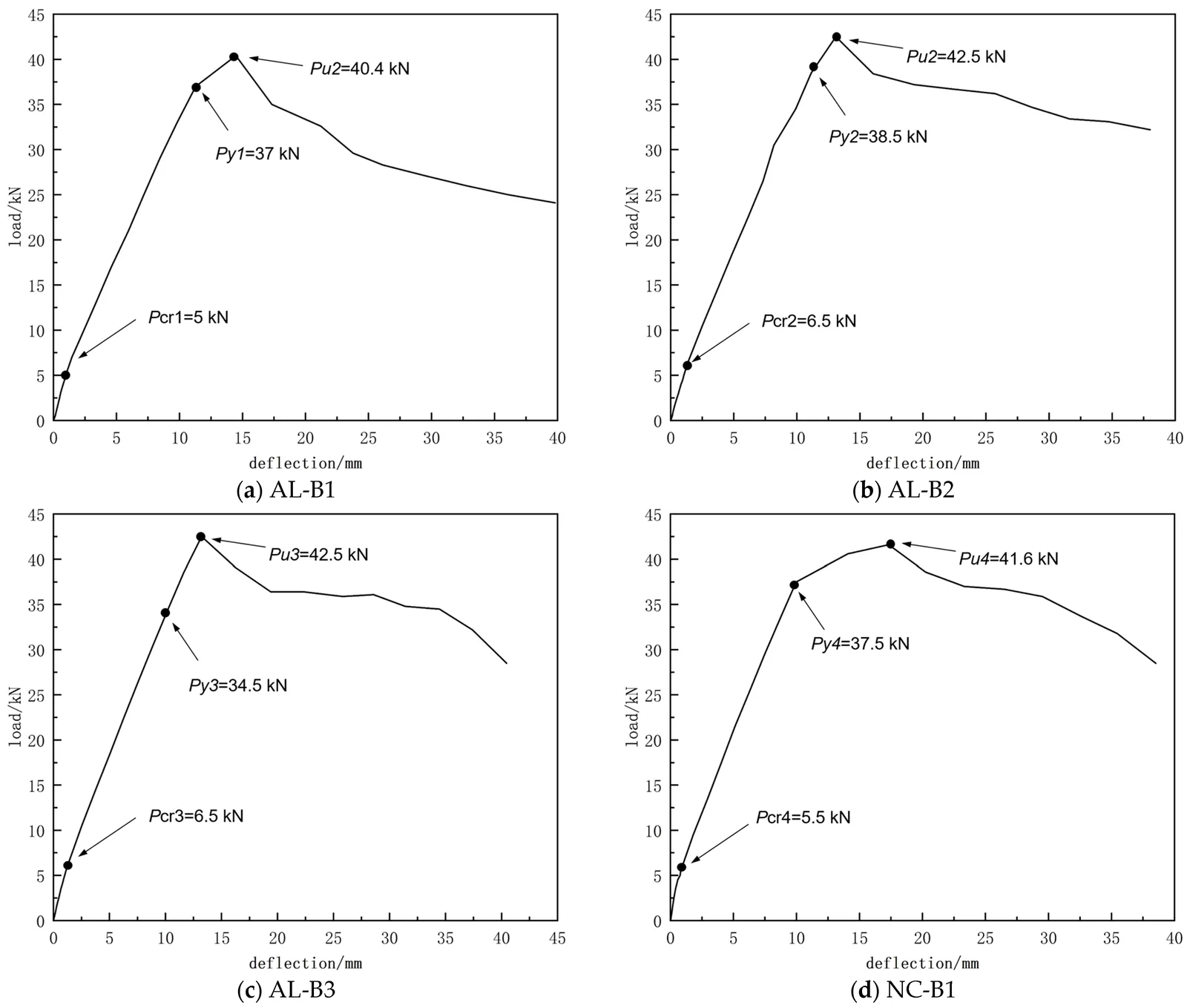
Midspan Load–Deflection Curves of Test Beams. Note: *P_cr_*—cracking load; *P_y_*—load at steel reinforcement yielding; *P_u_*—peak load.

The midspan load–deflection curves of the four test beams are shown in Figure 7a–d. All four curves exhibited distinct mechanical stages from the initial elastic response to post-peak deformation. The peak loads were reached at midspan deflections ranging from 13.10 to 17.32 mm. After the peak loads were reached, the beams continued to deform under displacement-controlled loading, and the maximum recorded midspan deflections at test termination ranged from 38.01 to 40.45 mm.

In the elastic stage, the load–deflection curves exhibited approximately stable slopes, and the measured strains increased approximately linearly with load, indicating a stable elastic response. When the cracking load was reached, an inflection point appeared in the load–deflection curve together with a noticeable increase in deflection, corresponding to the first visible cracking of the tensile-zone concrete. As loading continued toward the yield load, the tensile reinforcement yielded, the slope of the load–deflection curve decreased substantially, and the beam stiffness decreased accordingly. After the peak load was reached, the curves entered the post-peak stage, during which the load gradually decreased while the deflection continued to increase. Under displacement-controlled loading, the midspan deflections reached approximately 40 mm at test termination. The tests were terminated after severe crushing and spalling developed in the concrete compression zone. At the final recorded points, AL-B1, AL-B2, AL-B3, and NC-B1 still carried 24.1, 32.2, 28.5, and 28.5 kN, respectively, showing that the beams retained post-peak residual load-carrying capacity at test termination.

To facilitate a direct comparison among the four test beams, the principal flexural test results are summarized in Table 4.

**Figure 8:**
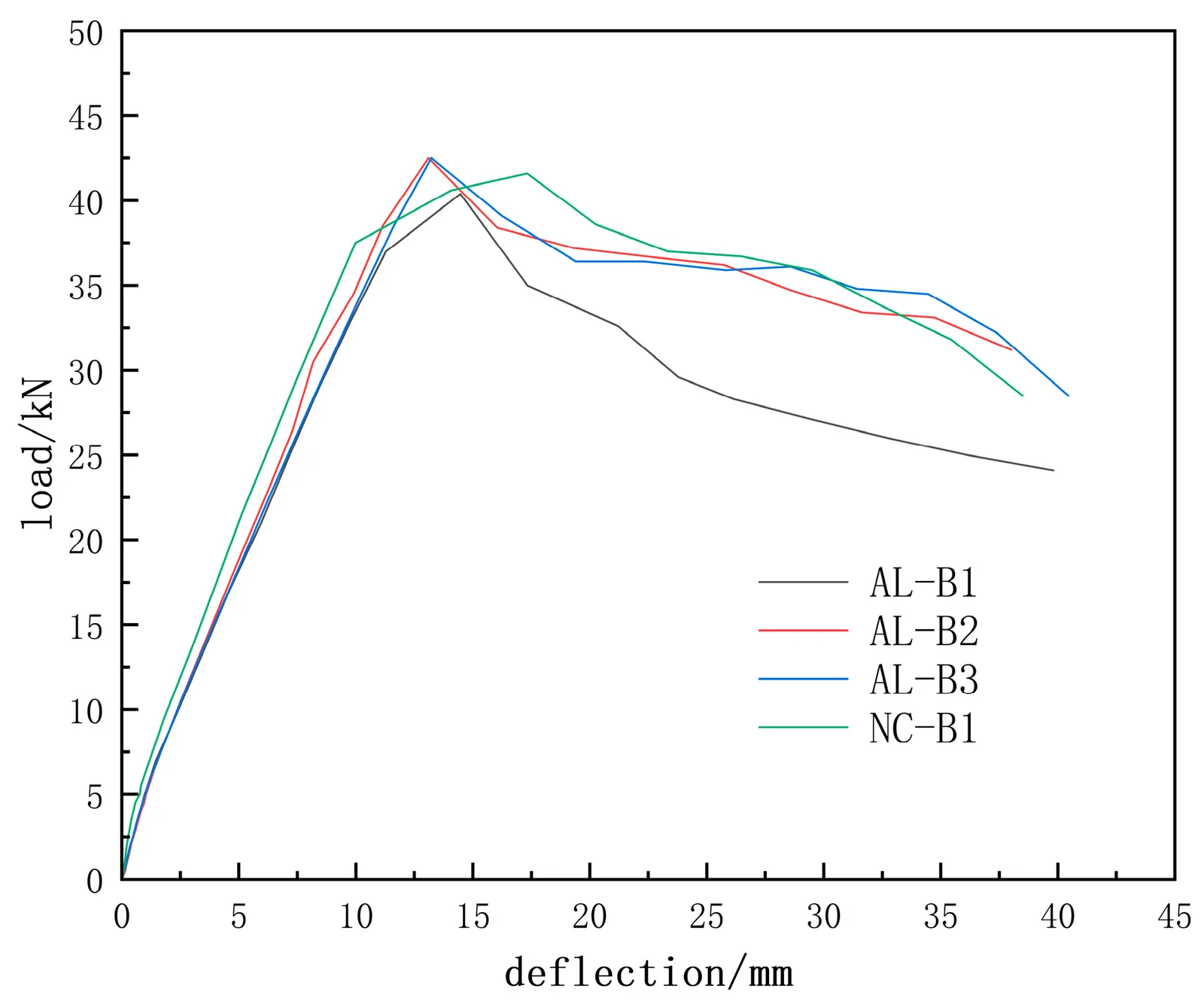
Summary of Load–Midspan Deflection Curves for Specimens.

Comparative analysis of the load–deflection curves of the four test beams (Figure 8) further clarifies the similarities and differences in the mechanical responses of the two beam types. Before cracking, the three ALSCC beams exhibited elastic-stage load–deflection stiffnesses ranging from 4.69 to 5.31 kN/mm, whereas NC-B1 exhibited a higher stiffness of 6.68 kN/mm. The average elastic-stage stiffness of the ALSCC beams was 4.93 kN/mm, approximately 26% lower than that of NC-B1. Because the static elastic moduli of the two concretes were not directly measured in the present study, this difference is reported as an experimental observation and is not attributed solely to the aggregate type. The three ALSCC beams exhibited peak loads of 40.4–42.5 kN, while the peak load of NC-B1 was 41.6 kN. Thus, comparable peak flexural resistance was observed among the four tested beams. However, because the two concretes differed in their measured material properties, the observed beam-level differences should not be attributed solely to aggregate type.

After the peak load, all four beams exhibited a decrease in load with increasing deflection, although some specimen-to-specimen differences were observed in the post-peak responses. Nevertheless, tensile-reinforcement yielding had already occurred before crushing of the compression-zone concrete in all tested beams. Therefore, the observed differences in post-peak response did not change the under-reinforced flexural failure sequence of the tested beams.

At test termination, the maximum recorded midspan deflections of all four beams were close to 40 mm, and the load–deflection curves showed continued deformation during the post-peak stage. Together with the substantially lower density of ALSCC, the specimen-level results observed in the present test program suggest that ALSCC may have potential for self-weight-sensitive flexural members; however, broader engineering applicability requires further experimental validation.

Within the present test program, the three ALSCC beams exhibited peak loads within the range of 40.4–42.5 kN, while the peak load of NC-B1 was 41.6 kN. However, because only one normal-concrete control beam was tested, the comparison with NC-B1 should be regarded as a specimen-level reference rather than a statistically representative comparison between the two concrete types. The limited beam sample size does not permit statistical generalization of the observed differences.

### 3.2. Sectional Strain Distribution and Plane-Section Assumption

**Figure 9:**
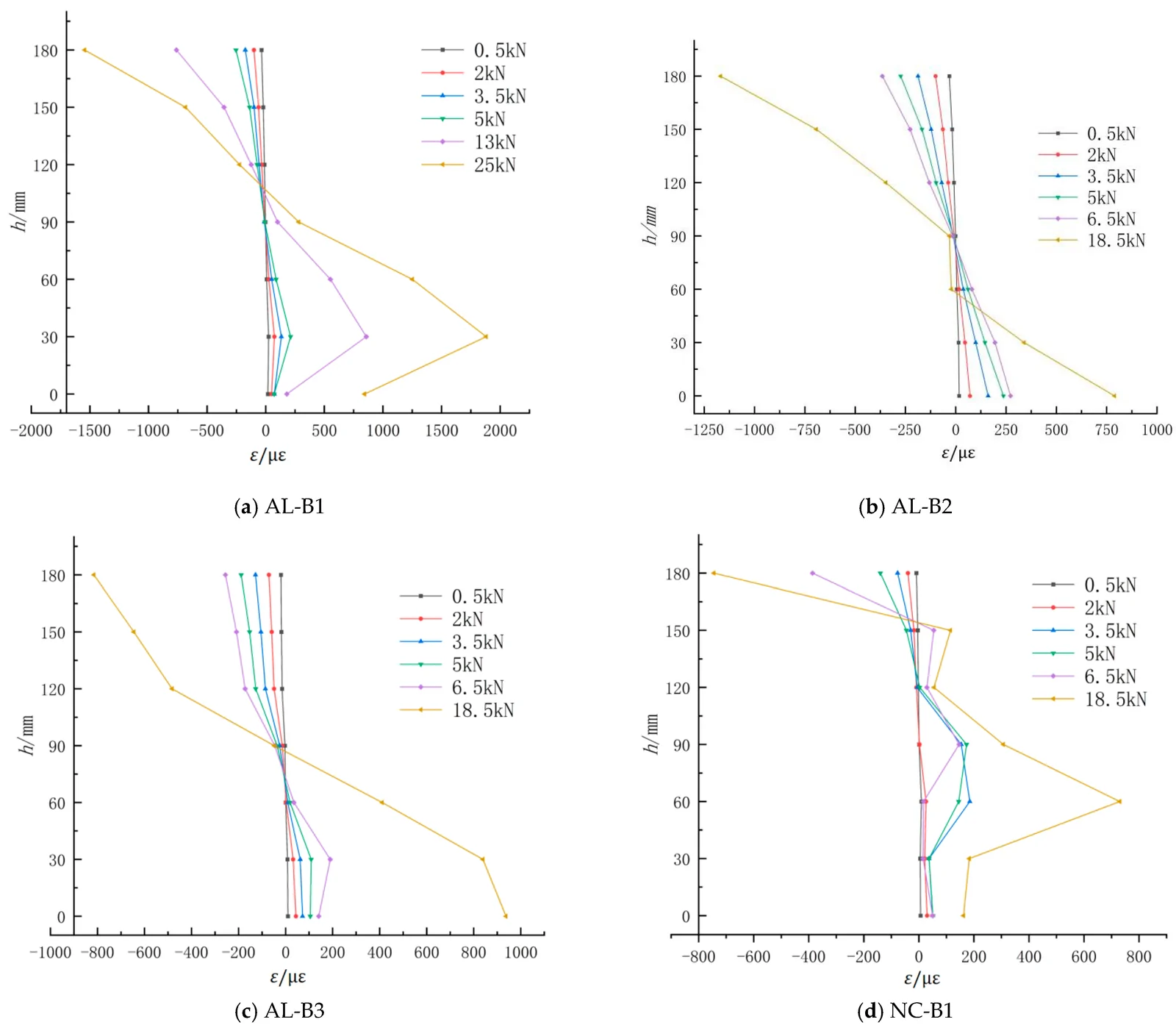
Strain Measurement Diagram of Test Beams Along Section Depth.

According to the strain profiles shown in Figure 9, the sectional strain distributions of the tested beams were approximately linear over the investigated load range. For AL-B1 under a high load of 25 kN, the strain transitioned uniformly from tensile strain at the beam bottom to compressive strain at the beam top, and the profile was approximately linear. For AL-B2 and AL-B3 at 18.5 kN, the strain profiles under different loads also showed an approximately linear distribution. Even after cracking, the measured strain profiles of the ALSCC beams remained approximately linear within the investigated load range. For NC-B1, the strain profiles were also generally regular at lower load levels, while somewhat larger deviations from linearity were observed as the load increased, particularly at 18.5 kN. This behavior suggests that, after cracking, the contribution of the tensile-zone concrete to the sectional tensile response decreased more noticeably in NC-B1.

The measured strain distributions suggest that both the ALSCC beams and NC-B1 generally conformed to the plane-section assumption before steel yielding. Within the present test program, the ALSCC specimens showed relatively stable strain distributions after cracking; however, because only one normal-concrete control beam was tested, the present comparison does not support a general conclusion that ALSCC beams exhibit superior plane-section behavior. In the elastic stage, the strain curves of all beams were close to ideal straight lines, and the neutral axis remained approximately near mid-depth.

The observed differences may be related to the different aggregate characteristics and crack-development mechanisms of the two concrete types. Within the measured load range, the ALSCC beams maintained relatively stable sectional strain distributions after cracking. Further tests with additional control specimens are required to determine whether this behavior represents a general characteristic of ALSCC beams.

### 3.3. Reinforcement-Strain Response

**Figure 10:**
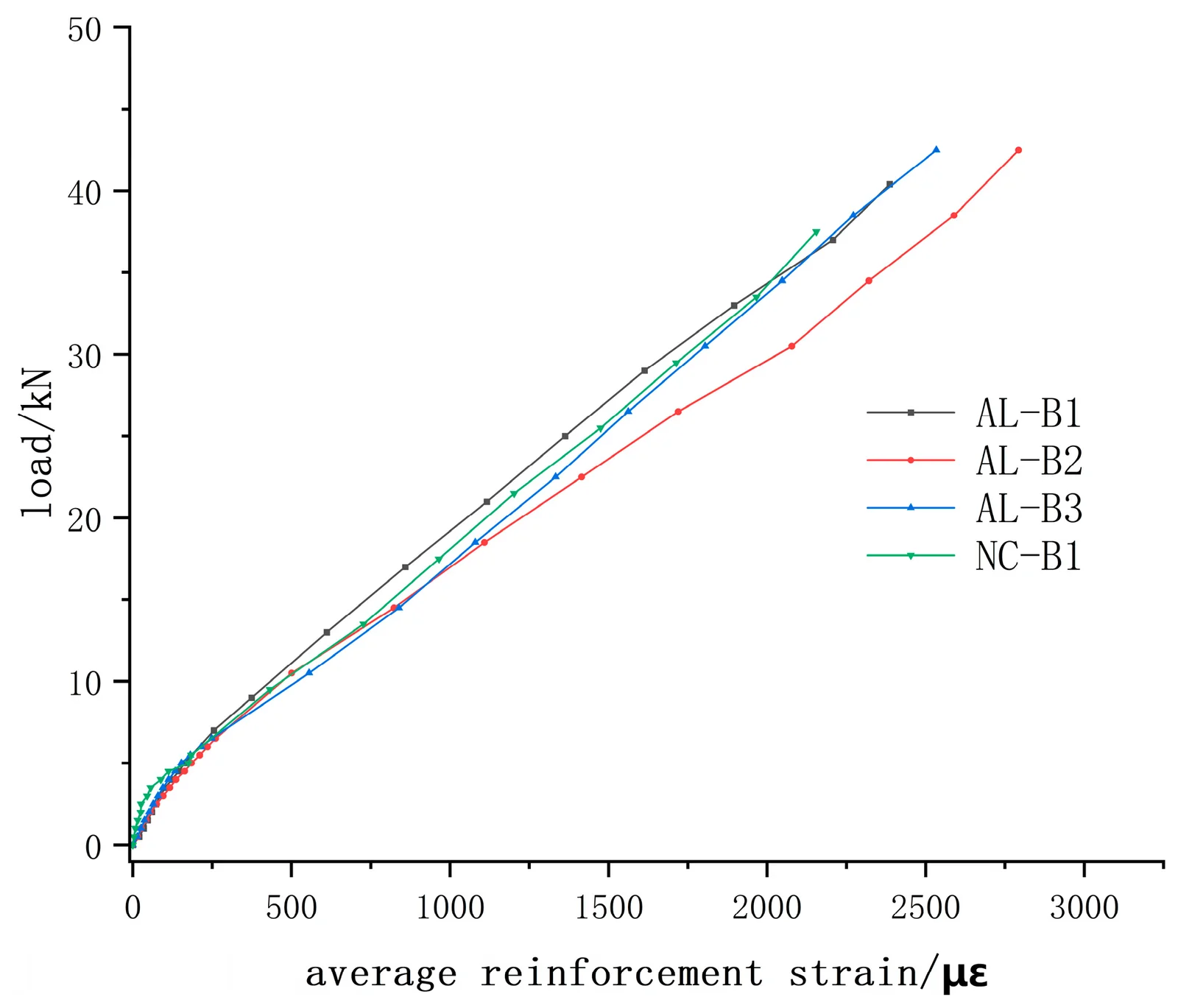
Load-Average Reinforcement-Strain Curves.

As shown by the load–average reinforcement-strain curves in Figure 10, the reinforcement-strain responses of the three ALSCC beams were broadly comparable to the response of NC-B1 within the measured range. During the elastic stage, the four curves were close to each other, and the load increased approximately linearly with reinforcement strain.

After yielding of the tensile reinforcement, the overall slopes of the curves remained similar. At higher reinforcement-strain levels, the ALSCC beams sustained load levels broadly comparable to those of NC-B1, although some specimen-to-specimen differences were observed.

Overall, the reinforcement-strain responses of the tested ALSCC beams were broadly comparable to the response of NC-B1, and no pronounced abnormality in the reinforcement-strain response was observed within the present test program. However, bond stress, reinforcement slip, anchorage performance, and local strain transfer along the reinforcement were not directly measured. Therefore, the present results should be interpreted only in terms of reinforcement-strain response and should not be regarded as direct evidence of improved steel–concrete bond performance or delayed bond slip. Direct bond tests, end-slip measurements, or reinforcement-strain measurements at multiple locations along the bar would be required to evaluate bond behavior quantitatively.

## 4. Conclusions

This study investigated LC30 all-light shale ceramsite concrete (ALSCC) and C30 normal concrete. Cubic specimen material-property tests and comparative flexural tests on three ALSCC beams (AL-B1, AL-B2, and AL-B3) and one normal-concrete control beam (NC-B1) were carried out to investigate the physical, mechanical, and flexural behavior of ALSCC and to provide preliminary experimental evidence under the present test conditions. The main conclusions are summarized as follows:

(1) ALSCC exhibited a substantially lower density than normal concrete. Its average density was 65.2% of that of normal concrete, while its cube compressive strength and splitting tensile strength were 80.6% and 82.3% of those of normal concrete, respectively. The splitting-tensile-strength-to-compressive-strength ratios of the two concretes were broadly comparable.
(2) All ALSCC beams exhibited typical under-reinforced flexural failure, with yielding of the tensile reinforcement preceding crushing of the concrete in the compression zone. Their cracking loads ranged from 5.0 to 6.5 kN, and their peak loads ranged from 40.4 to 42.5 kN. The corresponding midspan deflections at peak load ranged from 13.10 to 14.47 mm. Under continued displacement-controlled loading, maximum midspan deflections of 38.01–40.45 mm were recorded at test termination. The ALSCC beams exhibited a relatively distributed visible crack pattern during loading.
(3) Within the measured load range, the sectional strain distributions of the tested ALSCC beams were generally consistent with the plane-section assumption before steel yielding. The average elastic-stage stiffness of the three ALSCC beams was 4.93 kN/mm, approximately 26% lower than that measured for NC-B1. Relatively stable sectional strain distributions were observed after cracking; however, these observations should be interpreted within the limited scope of the present test program.
(4) The load–reinforcement-strain responses of the tested ALSCC beams were broadly comparable to the response of the normal-concrete control beam. No pronounced abnormal reinforcement-strain response was observed within the present tests. Since bond stress and reinforcement slip were not directly measured, the present results are interpreted only in terms of reinforcement-strain response and do not constitute direct evidence of improved steel–concrete bond performance.
(5) Within the scope of the present test program, the ALSCC beams exhibited peak flexural resistance comparable to that of the normal-concrete control beam, while the density of ALSCC was substantially lower than that of normal concrete. However, because the two concrete systems differed in their measured mechanical properties, the observed beam-level differences should not be attributed solely to aggregate type. These observations suggest that ALSCC may have potential for flexural members where structural self-weight is an important consideration; however, broader engineering applicability requires further experimental validation.

Given the limited scale of the present experimental program, particularly the use of three ALSCC beams and one normal-concrete control beam, the above conclusions should be regarded as preliminary and specific to the material composition, specimen geometry, reinforcement arrangement, and loading conditions adopted in this study. Further tests with larger sample sizes, additional control specimens, direct measurement of concrete elastic modulus, and a wider range of design parameters are needed to assess the repeatability and general applicability of the observed behavior.

Future work should examine the shear resistance and long-term performance of ALSCC flexural members. Further studies on reinforcement optimization and material modification may help clarify approaches for mitigating brittleness and provide additional evidence for evaluating the engineering applicability of ALSCC.

## Figures and Tables

**Table 1:** Mix proportions and measured slump of ALSCC and C30 normal concrete.

Constituent/Property	Unit	ALSCC	C30 Normal Concrete
P·O 42.5 ordinary Portland cement	kg/m^3^	370	340
Water	kg/m^3^	200	170
Coarse aggregate	kg/m^3^	370, shale ceramsite	1050, crushed stone (5–20 mm)
Fine aggregate	kg/m^3^	540, shale ceramsite sand	760, river sand
Cellulose ether	kg/m^3^	0.3	—
Water-reducing admixture	kg/m^3^	7.0	4.0
Water-to-cement ratio	–	0.54	0.50
Slump	mm	145	160

**Table 2:** Measured physical and mechanical properties of concrete specimens.

Specimen No.	Density (kg/m^3^)	Failure Load (kN)	Strength (MPa)
NC-C1	2315.56	813.78	36.17
NC-C2	2355.41	819.12	36.41
NC-C3	2359.11	791.12	35.19
NC-C4	2245.19	834.96	37.11
NC-C5	2283.41	937.73	41.68
NC-C6	2286.37	942.89	41.91
NC-S1	2305.19	100.73	2.85
NC-S2	2338.37	103.10	2.92
NC-S3	2327.71	108.65	3.08
NC-S4	2270.08	113.68	3.22
NC-S5	2280.30	133.83	3.79
NC-S6	2297.34	110.32	3.12
AL-C1	1472.74	712.44	31.66
AL-C2	1494.37	635.34	28.24
AL-C3	1523.56	723.32	32.14
AL-S1	1525.48	99.50	2.82
AL-S2	1496.30	85.28	2.41
AL-S3	1509.48	90.74	2.57

**Table 4:** Summary of principal flexural test results of the beams.

Beam	*P_cr_* (kN)	*P_y_* (kN)	*P_u_* (kN)	*δ_u_* (mm)	*δ_t_* (mm)	*K_e_* (kN/mm)
AL-B1	5.0	37.0	40.4	14.47	39.81	5.31
AL-B2	6.5	38.5	42.5	13.10	38.01	4.69
AL-B3	6.5	34.5	42.5	13.22	40.45	4.78
NC-B1	5.5	37.5	41.6	17.32	38.51	6.68

Note: $P_{cr}$ is the cracking load; $P_{y}$ is the load at tensile-reinforcement yielding; $P_{u}$ is the peak load; $\delta_{u}$ is the midspan deflection at peak load; $\delta_{t}$ is the maximum recorded midspan deflection at test termination; and $K_{e}$ is the elastic-stage load–deflection stiffness determined from the linear regression slope of the load–midspan deflection response up to the cracking load. All four beams exhibited under-reinforced flexural failure.

## Data Availability

The original contributions presented in this study are included in the article. Further inquiries can be directed to the corresponding author.

## References

[1] Gao C., Li Z., Ma H., Li M., Shi N., Tian S. (2025). Study on the Flexural Performance of Composite Wall Panels with Ceramsite Foam Concrete and Normal Concrete. Buildings.

[2] Zhao X. (2025). Experimental Study on Static and Dynamic Mechanical Properties and Microstructure of Shale Ceramsite Concrete. Master’s Thesis.

[3] Huang Y., Huang W., Huang Q., Tuo W., Feng Q. (2025). Study on the Axial Compressive Behavior and Constitutive Relationship of Lightweight Mixed Ceramic Concrete. Materials.

[4] Zhao Z., Pu Z., Lin C., Xiao J., Yao L., Ji C., Liu Y. (2024). Research Progress on Properties of Unburned Ceramsite and Ceramsite Concrete. Mater. Rep..

[5] Gu F., Li C., Wang X., Yang Y., Liu H. (2025). Experimental Study on the Compressive Behavior of Fiber-Reinforced Ceramsite Concrete. Materials.

[6] Li W., Liu X., Li J., Shao J., Yang M. (2025). Research on Performance of Basalt Fiber Reinforced Lightweight Ceramsite Cement Concrete. Henan Sci. Technol..

[7] Xiao Y. (2025). Research on Mechanical Properties of Ceramsite Concrete Modified by Nano-Silica. Liaoning Chem. Ind..

[8] Li M., Xu D., Wang X. (2023). Experimental Study on Uniaxial Compressive Performance of Shale Ceramsite Concrete. J. Wuhan Univ. Technol..

[9] Xu Y., Bai Z., Lv Z., Zhou Y., Lu Y., Han J. (2026). Study on Mechanical Properties and Thermal Conductivity of Lightweight Ceramsite Concrete. Brick Tile.

[10] Ma Li C., Liu J., Wang Y. (2025). Influence of Ceramic Lightweight Aggregate on Concrete Performance. Concr. Cem. Prod..

[11] Li P., Liu H., Li J., Li J. (2024). Experimental Study on Mechanical and Durability Properties of Double-Steel Fiber Lightweight High-Strength Concrete. China Harb. Eng..

[12] Huang W., Zhao Y., Zhu A. (2019). Experimental Study on Short-Term Crack Development of High-Strength Reinforced Lightweight Aggregate Concrete Beams. J. Build. Struct..

[13] Du C., Tian W.L., Wang X.W., Wang D.J. (2012). Experimental Research on Ceramsite Concrete Beams. Appl. Mech. Mater..

[14] Zhou P., Tang Y., Yang W., He Z., Mo Z. (2019). Experimental Study on Flexural Bearing Capacity of Shale Ceramsite Concrete Beams. Bull. Chin. Ceram. Soc..

[15] Liang T., Huang L., Ding Y., Cui C., Li T. (2023). Experimental Study on Flexural Performance of Autoclaved Silicate Ceramsite Concrete Beams. Concr. Cem. Prod..

[16] (2019). Standard for Test Methods of Physical and Mechanical Properties of Concrete.

[17] (2021). Technical Specification for Lightweight Aggregate Concrete.

